# Elevated collagen-I augments tumor progressive signals, intravasation and metastasis of prolactin-induced estrogen receptor alpha positive mammary tumor cells

**DOI:** 10.1186/s13058-017-0801-1

**Published:** 2017-01-19

**Authors:** Craig E. Barcus, Kathleen A. O’Leary, Jennifer L. Brockman, Debra E. Rugowski, Yuming Liu, Nancy Garcia, Menggang Yu, Patricia J. Keely, Kevin W. Eliceiri, Linda A. Schuler

**Affiliations:** 1 Cellular and Molecular Biology Program, University of Wisconsin–Madison, Madison, WI USA; 2 Department of Comparative Biosciences, University of Wisconsin–Madison, Madison, WI USA; 3 Department of Cell and Regenerative Biology, University of Wisconsin–Madison, Madison, WI USA; 4 Department of Biostatistics & Medical Informatics, University of Wisconsin–Madison, Madison, WI USA; 5 Laboratory for Optical and Computational Instrumentation, University of Wisconsin–Madison, Madison, WI USA; 6 University of Wisconsin Paul P. Carbone Comprehensive Cancer Center, University of Wisconsin–Madison, Wisconsin, USA

**Keywords:** Tumor microenvironment, Collagen, Desmoplasia, Breast cancer, Extracellular matrix, Tumor progression, Prolactin

## Abstract

**Background:**

The development and progression of estrogen receptor alpha positive (ERα+) breast cancer has been linked epidemiologically to prolactin. However, activation of the canonical mediator of prolactin, STAT5, is associated with more differentiated cancers and better prognoses. We have reported that density/stiffness of the extracellular matrix potently modulates the repertoire of prolactin signals in human ERα + breast cancer cells in vitro: stiff matrices shift the balance from the Janus kinase (JAK)2/STAT5 cascade toward pro-tumor progressive extracellular regulated kinase (ERK)1/2 signals, driving invasion. However, the consequences for behavior of ERα + cancers in vivo are not known.

**Methods:**

In order to investigate the importance of matrix density/stiffness in progression of ERα + cancers, we examined tumor development and progression following orthotopic transplantation of two clonal green fluorescent protein (GFP) + ERα + tumor cell lines derived from prolactin-induced tumors to 8-week-old wild-type FVB/N (WT) or collagen-dense (c*ol1a1* ^*tm1Jae/+*^) female mice. The latter express a mutant non-cleavable allele of collagen 1a1 “knocked-in” to the *col1a1* gene locus, permitting COL1A1 accumulation. We evaluated the effect of the collagen environment on tumor progression by examining circulating tumor cells and lung metastases, activated signaling pathways by immunohistochemistry analysis and immunoblotting, and collagen structure by second harmonic generation microscopy.

**Results:**

ERα + primary tumors did not differ in growth rate, histologic type, ERα, or prolactin receptor (PRLR) expression between *col1a1* ^*tm1Jae/+*^ and WT recipients. However, the *col1a1* ^*tm1Jae/+*^ environment significantly increased circulating tumor cells and the number and size of lung metastases at end stage. Tumors in *col1a1* ^*tm1Jae/+*^ recipients displayed reduced STAT5 activation, and higher phosphorylation of ERK1/2 and AKT. Moreover, intratumoral collagen fibers in *col1a1* ^*tm1Jae/+*^ recipients were aligned with tumor projections into the adjacent fat pad, perpendicular to the bulk of the tumor, in contrast to the collagen fibers wrapped around the more uniformly expansive tumors in WT recipients.

**Conclusions:**

A collagen-dense extracellular matrix can potently interact with hormonal signals to drive metastasis of ERα + breast cancers.

**Electronic supplementary material:**

The online version of this article (doi:10.1186/s13058-017-0801-1) contains supplementary material, which is available to authorized users.

## Background

Metastatic estrogen receptor α positive (ERα+) breast cancer is the leading cause of breast cancer mortality [1–3]. However, the mechanisms that drive progression of these cancers are poorly understood, in part because there are few animal models of ERα + breast cancer. The extracellular matrix (ECM) is increasingly recognized as an important contributor to tumor behavior. Aggressive tumors frequently display desmoplasia, one component of which is increased deposition of fibrillar collagens such as collagen-I [4–6]. This increased matrix deposition frequently increases the stiffness of the tumor and adjacent tissue [7]. Stiff ECM environments drive tumor-progressive characteristics both in vitro [8] and in mouse models [9–11]. Moreover, tumors can actively remodel the surrounding ECM. Aligned collagen-I fibers increase ECM stiffness [12], and collagen fibers aligned perpendicularly to the boundary of larger tumors predict poor outcomes, particularly of ERα + cancers [13]. Collagen-I density/stiffness increases pro-tumorigenic signaling cascades in tumor epithelia, such as focal adhesion kinase (FAK), src family kinases (SFKs), and extracellular regulated kinase (ERK)1/2 [14]*.* The effects of these changes on hormonal signals and consequences for their roles in the progression of ERα + tumors are not well-understood.

Large prospective epidemiologic studies have linked the hormone, prolactin (PRL), to increased risk of development of aggressive ERα + cancers, and smaller-scale studies also suggest that it contributes to their progression [15–18]. However, activation of STAT5, the primary physiological effector of prolactin (PRL), is associated with favorable clinical outcomes [19–21], and reduces invasion of breast cancer cells in vitro [22, 23]. Interestingly, FAK, SFKs, and ERK1/2 are also activated by PRL [24–26], and the ability of PRL to activate STAT5 is inversely related to its ability to activate AP-1 via mitogen-activated protein (MAP) kinases and augment invasiveness [27]. We recently reported that collagen-I density/stiffness is a major determinant of the signaling pathways that are available to the PRL receptor (PRLR). Whereas ERα + breast cancer cells cultured in low density/compliant three-dimensional collagen I matrices respond to PRL predominantly by activating physiological JAK2/STAT5 signals, high density/stiff matrices shift PRL responses to pathological ERK1/2 signals and increase invasiveness [28]. Under these latter conditions, PRL crosstalk with estrogen increases alignment of the matrix perpendicular to the tumor edge [29], similar to that correlated with decreased survival of patients with ERα + tumors [13, 30]. These data indicate that PRL and the ECM cooperate to drive processes leading to progression of breast cancer. However, examination of this interplay in vivo is necessary to confirm its importance and investigate clinical applications.

In order to examine the interaction between PRL and increased collagen-I deposition in an immunocompetent environment in vivo, we took advantage of well-characterized genetically modified mouse models. Hormonally responsive mouse models of breast cancer are rare [31, 32]. The neu-related lipocalin-prolactin (NRL-PRL) transgenic mouse mimics the local PRL synthesis in the mammary glands of women. Nulliparous female mice spontaneously develop aggressive mammary tumors, about 75% of which are ERα + [33]. ERα + tumor cell lines derived from these adenocarcinomas are readily transplantable to syngeneic recipients [34]. To model increased collagen I, we utilized the *Col1a1* ^*tmJae1*^mouse [35]. Mutation of the MT1-MMP cleavage site in Col1a1 reduces collagen-I turnover, leading to its accumulation, without a need for additional fibrotic factors and any associated confounding activities. We have previously shown that this increases metastasis from experimental MMTV-PyMT-induced mammary tumors, and results in a collagen fiber signature that predicts poor survival in patients with breast cancer [13, 14, 36].

Here, we orthotopically transplanted clonal green fluorescent protein (GFP)-labeled PRL-induced ERα + mammary tumor cell lines into syngeneic wild-type (WT) or heterozygous mutant collagen-I female mice (*Col1a1* ^*tmJae/+*^, mCol1a1). Tumors that developed in the mCol1a1 environment had similar rates of growth, morphology, ERα, and PRLR expression to those in the WT collagen environment. However, the mCol1a1 environment increased circulating tumor cells (CTCs), and the number and size of lung metastases. It also altered the pattern of activated signaling cascades in the primary tumors: tumors in mCol1a1 female mice had lower pSTAT5 and increased pERK1/2 and pAKT expression, consistent with predictions from in vitro studies. Moreover, the alignment of intratumoral collagen fibers near the tumor boundary in mCol1a1 recipients was more perpendicular to the tumor edge and oriented in parallel to protrusions invading into the adjacent fat pad. These data indicate that extracellular matrix can potently interact with hormonal signals to drive the development of metastasis from ERα + breast cancers.

## Methods

### Reagents

Antibodies used in these studies were purchased from the following vendors: ERK1/2 (#9102), pERK1/2 (#9101), AKT (#9272), pAKT S473 (#9271), pAKT S473 (#3787S, for immunohistochemical analysis) from Cell Signaling Technology (Danvers, MA, USA); pSTAT5 (#71-6900) from Invitrogen (Grand Island, NY, USA); ERα (#sc-542), PRLR (#sc-20992), STAT5 (#sc-835x) from Santa Cruz Biotechnology (Santa Cruz, CA, USA); eGFP (#AB6658) from AbCam (Cambridge, MA, USA); PR (#A0098) from Dako (Carpinteria, CA, USA); biotinylated goat anti-rabbit (#BA-100) from Vector Labs (Burlingame, CA, USA); pan-actin (#125-ACT) from Phosphosolutions (Aurora, CO, USA); APC-conjugated CD31 (#551262) and CD45 (#559864) from BD Biosystems (San Jose, CA, USA). Avidin-biotin complex (ABC) (#PK-4000) and ImmPACT DAB (#SK-4105) were purchased from Vector Labs (Burlingame, CA, USA). All other reagents were obtained from Fisher Scientific or Sigma-Aldrich.

### Cell lines and culture

ERα + mouse mammary tumor cell lines were derived from a NRL-PRL mammary tumor [34, 37]. Two independently derived cell lines were stably transfected with eGFP, TC2GR12 (TC2) and TC4GR5 (TC4), and clonal sublines were maintained on tissue culture plastic in Roswell Park Memorial Institute medium (RPMI) 1640 supplemented with 10% FBS, 1% penicillin/streptomycin, and 1 mg/ml (TC2GR12) or 400 μg/ml (TC4GR5) puromycin as a selection factor. As shown in Additional file 1, TC2GR12 (TC2) and TC4GR5 (TC4) both express the rPRL transgene and exhibit somewhat differing levels of hormone receptor and signal effector proteins.

### Animals

Mice heterozygous for *Col1a1* ^*tmJae*^ [35] (*Col1a1* ^*tmJae/+*^ *;* mCol1a1) were backcrossed onto the FVB/N strain background for 10 generations. Mice were housed and cared for in accordance with the Guide for Care and Use of Laboratory Animals in AAALAC-accredited facilities. All procedures were approved by the University of Wisconsin-Madison Animal Care and Use Committee. For some experiments, 2.5 × 10^4^ (TC2GR12) or 7.5 × 10^4^ (TC4GR5) cells in 50 μl of sterile PBS were orthotopically injected into the left caudal mammary fat pads of 8 to 10-week-old FVB/N WT or mCol1a1 female mice and allowed to progress to end stage (tumor 1.5 cm in diameter). All recipients survived to end stage. For analysis of early-stage tumors, cell lines were injected bilaterally into the caudal mammary fat pads of 8 to 10-week-old WT or heterozygous mCol1a1 female mice, and tumors were allowed to progress for 17 days (TC2) or 24 days (TC4), the time of peak CTCs, respectively, before collection. Each animal was palpated biweekly to assess tumor development, and tumor diameter was measured using electronic calipers. Tumor volume was calculated as the largest diameter * (smallest diameter^2^) * 0.4.

### Flow cytometry

Peripheral blood (100 μl) was collected from each animal weekly from a maxillary vein in 6 U heparin sulphate, starting 3 days after tumor cell transplantation. Red blood cells were lysed in 0.15 M NH_4_Cl + 1.2 mM EDTA for 10 minutes with gentle agitation. Cleared blood was centrifuged at 300 × g for 5 minutes, washed in PBS, and stained with anti-CD31/APC (1:100) and anti-CD45/APC (1:100) for 30 minutes on ice to label hematopoietic cells. The labeled cell suspension was then washed, fixed in 2% paraformaldehyde, and analyzed for endogenous GFP and CD31/45+ hematopoietic cells on a BD Fortessa flow cytometer, using the gating strategy presented in Additional file 2. Seven blood samples from non-injected female mice (five WT, two mCol1a1) served as controls for each experiment, and average background auto-fluorescence was subtracted from the experimental results.

### Immunohistochemical analysis and immunofluorescence

Immunohistochemical analyses were performed as previously described [38]. Briefly, tissues were fixed in 10% neutral buffered formalin, embedded in paraffin, and serial-sectioned. Deparaffinized tissues were hydrated in decreasing concentrations of ethyl alcohol (EtOH) and endogenous peroxidase activity quenched in 3% H_2_O_2_ in methanol (MeOH). Dilutions of the primary antibodies, antigen retrieval, and blocking conditions are shown in Additional file 3. Secondary antibody (1:250) and signal amplification (avidin-biotin complex (ABC)) were performed at room temperature prior to chromogen-detection with ImmPACT 3,3-diaminobenzidine (DAB) according to the manufacturer’s instructions. Tissues were counterstained with hematoxylin, dehydrated, and mounted for microscopic analysis. For immunofluorescence experiments, tissues were processed as described without performing peroxidase quenching. Alexa-488 conjugated streptavidin (1:100) was incubated at room temperature for 1 h prior to mounting with anti-fade media and subsequent epifluorescence imaging.

### Lung metastasis analysis

All animals were allowed to progress to end stage (tumor diameter 1.5 cm). At this time, lungs were collected and fixed in 10% neutral buffered formalin. After 24 h, surface nodules on all lung lobes were counted under a dissection microscope. The left lobe was then sectioned in four step-sections of 50 μm with two serial sections at each step. GFP immunofluorescence was performed as described to assess micrometastatic load. Total numbers of GFP+ lesions were counted per × 10 field of view (FOV) over seven FOVs per lung. The area of each lesion was calculated and the total sum of lesion area determined per FOV. Three independent lungs were analyzed for each cell line/genotype combination over two 50-μm steps, using ImageJ [39].

### Immunoblotting

Snap-frozen tumor pieces were homogenized in ice cold 40 mM Tris, 276 mM NaCl, 20% glycerol, 2% NP-40, 4 mM EDTA, 20 mM NaF, 2 mM sodium orthovanadate, 40 μg/ml phenylmethane sulfonylfluoride (PMSF), and 50 μg/ml aprotinin. Briefly, 30 μg of tumor homogenate was fractionated by SDS-PAGE, transferred to polyvinylidene fluoride (PVDF) membranes, and then probed with appropriate antibodies (ERK1/2, 1:2000; pERK1/2, 1:5000; STAT5, 1:50000; pSTAT5, 1:1000; ERα, 1:1000; AKT, 1:2000; pAKT S473, 1:1000; PRLR, 1:1000). Signals were visualized by enhanced chemiluminescence and quantified by densitometry (UVP Visionworks). All end-stage tumors were analyzed.

### Collagen imaging and analysis

Paraffin-embedded, 5-μm sections of early lesions were stained with picrosirius red as previously described [40]. Polarized light microscopy with a × 10 objective was utilized to assess birefringence that is specific to fibrillar collagens. Multiphoton microscopy and second harmonic generation (SHG) imaging were performed as previously described [41]. Briefly, collagen was visualized with a laser excitation of 890 nm with a 445 ± 20 nm emission filter to detect backwards SHG focused on the sample with a × 20/0.75NA objective. The overall fiber alignment analysis was performed with the CurveAlign software following fiber detection utilizing CT-FIRE [42, 43]. The alignment coefficient was based on the orientation of the collagen fibers in the same image and ranges from 0 to 1, where the larger the alignment coefficient, the more aligned the fibers.

### Statistical analysis

Statistical analyses were performed in GraphPad Prism v4.0 or SAS/STAT v9.4. Data were tested for normality using the Shaprio-Wilk test. Data were analyzed using the unpaired *t* tests (normal data) or Mann-Whitney *U* non-parametric test (non-normal data) as indicated. Tumor growth and CTC data were analyzed using two-way repeated measures analysis of variance (ANOVA), taking into account values across days in the same animals. Collagen alignment was quantified using repeated measure group comparison with the SAS Proc Mixed model to take into account image–image and tumor − tumor variation for each cell line/genotype combination. Significance of the data are presented as *p < 0.05, **p < 0.01, ***p < 0.001, or ****p < 0.0001.

## Results

### Increasing collagen-I does not alter primary tumor growth, yet increases circulating tumor cells

**Fig. 1:**
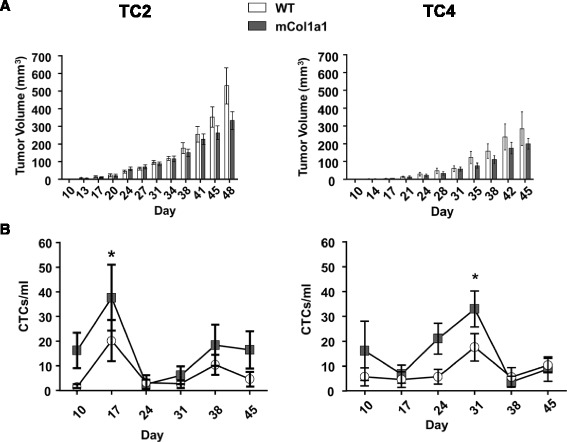
mCol1a1 does not alter the rate of tumor growth, but increases circulating tumor cells. **a**-**b** Tumor growth of tumor cell line (*TC*)2 (**a**) and TC4 (**b**) tumors until collection of the largest tumors began. Tumor volume was measured biweekly. Mean ± SEM TC2 animals: wild-type (*WT*) n = 7, mCol1a1 n = 6. TC4 animals: WT n = 7, mCol1a1 n = 8. Time significantly influenced tumor volume in both TC2 (**a**) and TC4 (**b**) tumors (*p* < 0.0001, two-way repeated measures ANOVA), but genotype did not. **c**-**d** Circulating tumor cells (*CTCs*) in peripheral blood of animals bearing TC2 (**c**) or TC4 (**d**) tumors. Peripheral blood was collected weekly and analyzed for green fluorescent protein positive (GFP+) CTCs as described in “Methods”. Mean ± SEM TC2 recipients: WT n = 7, mCol1a1 n =6. TC4 recipients: WT n = 7, mCol1a1 n =8. Genotype significantly altered levels of CTCs (TC2, *p* < 0.0050; TC4, *p* < 0.0320; repeated measure two-way ANOVA, taking into account values across days in the same animals). Levels of CTCs peaked when tumors were relatively small, regardless of genotype (TC2, day 17 is significantly different from all other days except day 38, *p < 0.05; TC4, day 31 is significantly higher than day 17 and day 38, *p < 0.05)

In order to study the effect of a collagen-dense environment on progression of PRL-induced ERα + mammary cancer, we orthotopically transplanted GFP+ TC2 or TC4 tumor cells into WT or mCol1a1 female mice and monitored growth until end stage. Increased collagen-I did not alter the growth of either TC2 or TC4 tumors (Fig. 1a-b). In order to assess the mobilization of tumor epithelia into the bloodstream as an indicator of metastatic potential, peripheral blood was collected weekly from the maxillary vein and analyzed for circulating GFP+ tumor cells (CTCs) as described in “Methods”. In mCol1a1 recipients of both TC2 and TC4 cells, CTCs were significantly higher than in WT recipients (Fig. 1b, TC2, *p* < 0.0050; TC4, *p* < 0.0320). Interestingly, levels of CTCs peaked considerably prior to end stage (TC2, day 17; TC4, day 31 after transplantation).

### Collagen-I does not change tumor histology or hormone receptor status

**Fig. 2:**
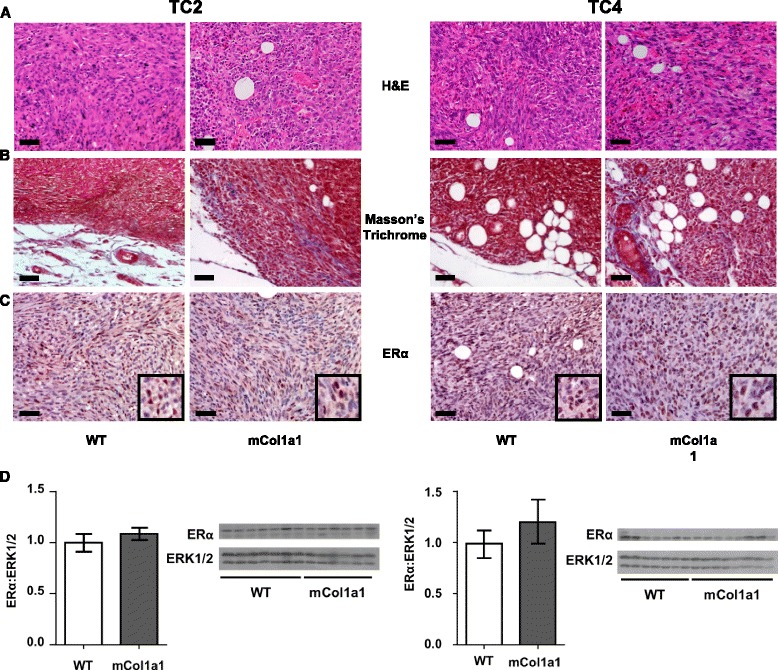
mCol1a1 does not alter tumor histologic type or hormone receptor status. Tumor cell line (*TC*)2 tumors (*left panel*) and TC4 tumors (*right panel*) were collected at end stage and processed for histological analysis as described in “Methods”. **a** Hematoxylin and eosin stain (*H&E*). **b** Masson’s Trichrome stain. **c** Estrogen receptor (*ER*)α immunohistochemical analysis. Original magnifications × 200; *scale bar* = 50 μm. **d** Immunoblotting of tumor lysates with the indicated antibodies. ERα levels were normalized to total extracellular regulated kinase (ERK)1/2. Note that increased intratumoral collagen-I in the mCol1a1 environment contributed to the protein harvested, reducing levels of signaling proteins when equal amounts of protein were loaded. Mean ± SEM TC2 tumors: wild-type (*WT*) n = 7, mCol1a1 n = 6; TC4 tumors: WT n = 7, mCol1a1 n = 8. Unpaired *t* test, *p* > 0.05

Transplantation of both TC2 and TC4 cell lines resulted in spindle cell carcinomas in both WT and mCol1a1 recipients (Fig. 2a). As expected, tumors in mCol1a1 animals showed increased Masson’s trichrome staining near the tumor boundary compared to those in WT mice, reflecting increased connective tissue in this mutated environment (Fig. 2b). Like the parent cell lines, TC tumors were also ERα+, and collagen deposition did not alter ERα expression discerned by either immunohistochemical (Fig. 2c) or western analyses (Fig. 2d). PRLR expression in these tumors was not altered by collagen accumulation (Additional file 4A); progesterone receptor (PR) was expressed in localized foci (Additional file 4B).

### Metastasis of ERα + mammary tumors is enhanced by increasing collagen-I

**Fig. 3:**
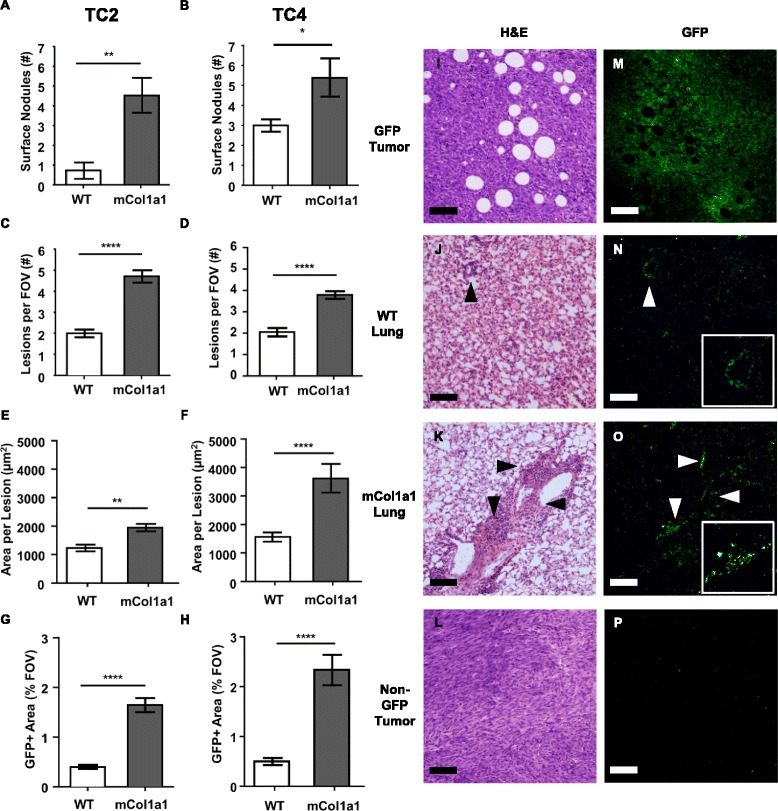
Metastasis of estrogen receptor (*ER*)α + tumors is enhanced by mCol1a1. **a**, **b** Nodules were counted on the surfaces of all lung lobes under dissection microscopy. Mean ± SEM tumor cell line (*TC*)2 tumors (**a**): wild-type (*WT*) n = 7, mCol1a1 n = 6. TC4 tumors (**b**): WT n = 7, mCol1a1 n = 8. Mann-Whitney U test, *p < 0.05, **p < 0.01. **c**-**h** Lungs were fixed and step-sectioned for histochemical analysis as detailed in “Methods”. Green fluorescent protein positive (*GFP*+) lesions were counted per × 10 field of view (*FOV*) (**c**, **d**). The area of GFP+ lesions was determined in ImageJ, and average size of individual lesions (**e**, **f**) and total lesion area per FOV (**g**, **h**) were calculated as described in “Methods”. Mean ± SEM Mann-Whitney *U* test. **p < 0.01, ****p < 0.0001. **i**-**o** Paired hematoxylin/eosin (*H&E*) and GFP stained sections from animals bearing TC4 tumors. Primary tumor (**i**, **m**), WT lung (**j**, **n**), mCol1a1 lung (**k**, **o**), and a non-GFP tumor control (**l**, **p**). *Arrowheads* indicate GFP+ lesions in lungs, one enlarged in each *inset*. Original magnifications × 100. *Scale bar* = 100 μm

ERα + breast cancers metastasize to multiple peripheral tissues, including the lungs [44]. As the mCol1a1 background increased CTCs (Fig. 1b), we collected the lungs at end stage to determine the consequences for metastasis to this site. Significantly more surface lung nodules were found in mCol1a1 recipients of both TC2 and TC4 cells (Fig. 3a, b; Additional file 5). Histologic examination revealed that mCol1a1 female mice bearing either TC2 or TC4 tumors also had higher numbers of metastatic lesions in the lung parenchyma (Fig. 3c, d) (*p* < 0.0001), visualized utilizing GFP as a marker (Fig. 3j, k, n, o). Moreover, these lesions were significantly larger (Fig. 3e, f) (*p* < 0.01), resulting in a greatly increased total metastatic burden in mCol1a1 mice (Fig. 3g, h) (*p* < 0.0001).

### mCol1a1 decreases pSTAT5, and increases pERK1/2 and pAKT in ERα + mammary tumors

We have previously shown that characteristics of the collagen ECM alter the spectrum of PRL-induced signals with behavioral consequences in human breast cancer cells in vitro: stiff/dense matrices reduce signals to STAT5, while increasing signals through ERK1/2 [28, 29]. While high pSTAT5 is associated with increased differentiation and lower proliferation [19–21], signals through ERK1/2 increase proliferation of breast cancer cells and may contribute to tumor progression [14, 45].

**Fig. 4:**
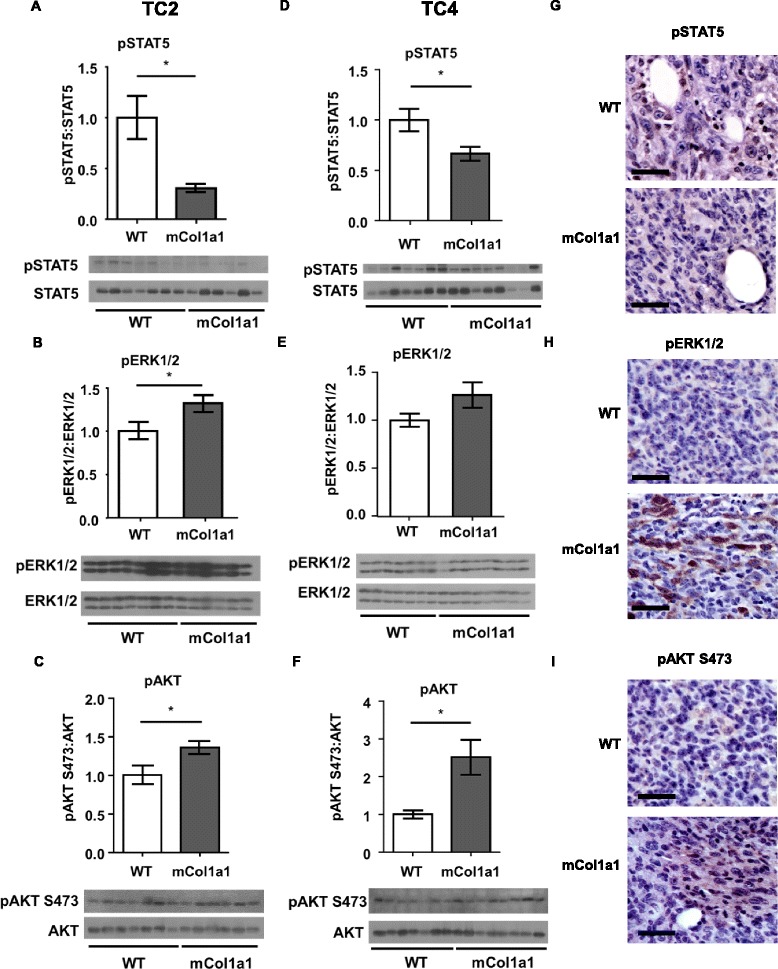
mCol1a1 decreases pSTAT5, and increases pERK1/2, pAKT. **a**-**f** TC2 (**a**-**c**) and tumor cell line (*TC*)4 (**d**-**f**) tumor lysates were analyzed by western blotting with the indicated antibodies. Mean ± SEM TC2 tumors: wild-type (*WT*) n = 7, mCol1a1 n = 6; TC4 tumors: WT n = 7, mCol1a1 n = 8. Unpaired *t* test, *p < 0.05. **g**-**i** Immunohistochemical analysis of pSTAT5 (**g**), pERK1/2 (**h**) and pAKT (**i**) in TC2 tumors. Original magnification × 200. *Scale bar* = 50 μm

Activation of AKT is associated with metastatic ERα + breast cancer, and the efficacy of anti-estrogens in combination with therapies targeting this pathway are currently under clinical evaluation [46, 47]. To determine if the high collagen-I environment in vivo alters these signaling cascades, we performed immunoblotting and immunohistochemical analyses. Tumors that grew from both cell lines in mCol1a1 recipients had lower pSTAT5 (*p* < 0.05, Fig. 4a, d), similar to human breast cancer cells cultured in dense/stiff matrices in vitro. TC2 tumors had higher pERK1/2 in mCol1a1 female mice, compared to WT recipients (*p* < 0.05, Fig. 4b), while TC4 tumors trended similarly (*p* = 0.11, Fig. 4e). Similarly, relative levels of pAKT were significantly higher in tumors from both cell lines (*p* < 0.05, Fig. 4c, f). These results were confirmed by immunohistochemical analysis, with lower nuclear pSTAT5 staining in tumors in mCol1a1 compared to WT recipients (Fig. 4g) (Additional file 6A), and higher nuclear and cytoplasmic pERK1/2 and pAKT in mCol1a1 female mice bearing TC2 (Fig. 4h, i) and TC4 (Additional file 6B, C) tumors.

### Early tumors in mCol1a1 recipients exhibit finger-like protrusions containing aligned collagen fibers

**Fig. 5:**
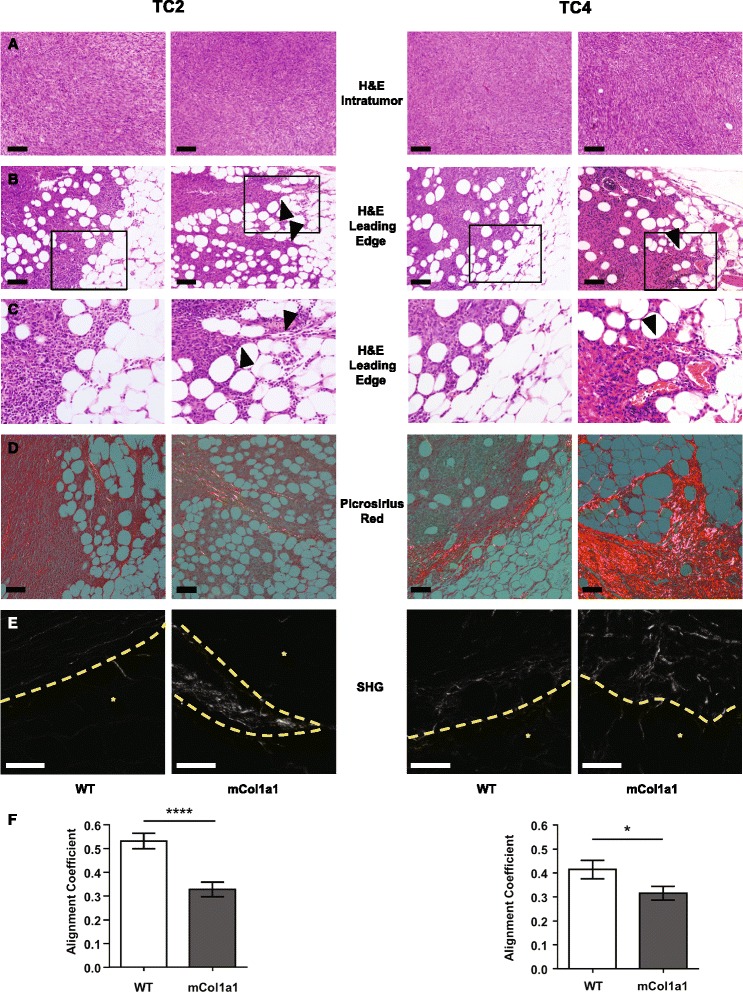
Early lesions in mCol1a1 recipients display invasive protrusions that contain aligned collagen fibers. **a** Hematoxylin/eosin stain (*H&E*) of tumor cell line (*TC*)2 tumors (*left panels*) at 17 days, TC4 tumors (*right panels*) at 24 days after transplantation. **b** Tumor edges (H&E). *Arrowheads* indicate finger-like protrusions into the mammary fat pad. **c** Higher magnification of boxed area from (**b**). **d** Picrosirius red staining. Note *yellow-orange* fibers in the protrusions in mCol1a1 tumors. Original magnification × 100. **a**, **b**, **d**, *scale bars* = 100 μm. **e** Second harmonic generation (*SHG*) imaging of collagen fibers. *Yellow dashed lines* indicate tumor boundaries. *Indicates adjacent fat pad. Representative images × 400 magnification. *Scale bars* = 50 μm. **f** CurveAlign analysis of relative collagen alignment following CT-FIRE fiber extraction as described in “Methods”. *0* = perfectly random fibers, *1* = perfectly aligned fibers. Mean ± SEM, n = 4 tumors of each cell line/host genotype combination, 7–9 images per tumor. Repeated measures, group comparison, SAS Proc Mixed model. *p < 0.05, ****p < 0.001. *WT* wild-type

At end stage, tumors nearly fill the mammary fat pads. In order to examine the characteristics of the tumor boundaries, we examined lesions earlier after transplantation, at the time when CTCs were elevated, particularly in mCol1a1 recipients (day 17, TC2 or day 24, TC4; Fig. 1b). Early-stage tumors in both WT and mCol1a1 recipients exhibited the spindle cell morphology evident at end stage (Fig. 5a). However, the tumor boundaries were quite different in the two host genotypes. Whereas both TC2 and TC4 tumors in WT recipients displayed a generally uniform pattern of expansion, lesions in mCol1a1 hosts exhibited many vascular finger-like protrusions into the surrounding mammary fat pads (Fig. 5b, c). Picrosirius red staining with polarized light microscopy revealed dense collagen fibers that were aligned with the tumor projections in mCol1a1 recipients, while fibers in tumors in WT animals were wrapped around the tumor edge (Fig. 5d), similar to collagen fibers observed in non-aggressive tumors [13, 48]. SHG microscopy in combination with analysis of the alignment of collagen fibers showed that collagen fibers in tumors of WT recipients aligned with one another parallel to the tumor edge, while those in tumors of mCol1a1 recipients were less consistently oriented (Fig. 5e, f) (*p* < 0.0001, TC2; *p* < 0.05, TC4), with many perpendicular to the bulk of the tumor and/or aligned with the protrusions into the fat pad.

## Discussion

Metastasis of ERα + breast cancer is poorly understood. ECM characteristics such as alignment of the collagen fibers in and around the tumor correlate with poor survival [13], demonstrating the importance of this component of the tumor microenvironment. However, the role of hormones in these events, and the consequences of changes in the ECM for hormone actions have not been clear. While some evidence supports a role for elevated PRL exposure in tumor progression [18], conflicting data on PRL-induced signal effectors suggest that other factors modulate PRL responses. We previously demonstrated in vitro that a dense/stiff collagen-I matrix shifts the balance of PRL signals from physiological to tumor progressive, and permits PRL and estrogen to reorient collagen-I fibers that mimic aggressive ERα + breast cancers in women [28, 29]. Here, we report that the predictions from these studies are confirmed in an immunocompetent mouse model in vivo: collagen-I accumulation promoted ERK1/2 and AKT activation and decreased STAT5 phosphorylation in PRL-induced ERα + mammary carcinomas, driving local invasion of the primary tumor, realigning collagen fibers, mobilizing tumor epithelia, and enhancing pulmonary metastases. These data indicate that the ECM can alter hormonal signals to drive aggressive behavior of ERα + tumors, providing mechanistic insight into their metastasis.

Extensive in vitro studies of human breast cancer cells have shown that the PRLR, like other cytokine receptors, can activate multiple signaling pathways [17]. However, the determinants of the spectrum of signaling pathways, and their respective roles in disease in vivo have been unclear. In normal mammary development, PRL signals primarily through JAK2/STAT5 to direct expansion and differentiation of the mammary epithelia in concert with ovarian steroids [49, 50]. Consistent with clinical data demonstrating that high STAT5 activation in breast cancers predicts favorable outcomes [19–21], constitutive activation of STAT5 in mouse models leads to well-differentiated tumors [51]. Interestingly, signals through this pathway are required for initiation, but not progression, of PRL-induced cancers in NRL-PRL females [52]. However, many ERα + cancers that develop in this model are very aggressive, and display highly activated ERK1/2 and AKT [33, 38], indicating activation of non-JAK2/STAT5 pathways. The data presented herein substantiate that the ECM is a potent determinant of the signaling cascades and outcomes of PRL actions in vivo, illuminating the apparent disparity between PRL exposure and activated STAT5 in the progression of clinical breast cancer. Moreover, our findings suggest that targeting non-canonical PRL signals may be of therapeutic benefit.

Mammographic density is a strong predictor of breast cancer risk [53–55]. However, epidemiologic data linking ECM density and breast cancer aggression are inconsistent [56, 57]. Mammographically dense tissue partly comprises increased fibrillar collagen [58]. Although this can stiffen the ECM [7, 59], the properties of density and stiffness are not always linked. For example, at involution following weaning, the mammary ECM contains higher levels of collagen I, yet the ECM is less stiff than that in the nulliparous gland [60]. This suggests a fundamental difference in ECM architecture between physiologic states and cancer which deserves further study. Interestingly, we have observed that the ECM features - stiffness and collagen-I ligand density - exert distinct effects on PRL-initiated signals, using polyacrylamide hydrogels in vitro: elevated density reduces PRL phosphorylation of STAT5, whereas stiffness augments PRL signals to ERK1/2 [61]. The alterations in both signaling cascades elicited by reduced collagen I degradation and the increase in collagen fibers aligned with invasive projections of the tumor mass observed in the current in vivo study suggest that PRL-expressing ERα + cancer can directly or indirectly remodel dense collagen matrices to increase stiffness and alignment. Further, PRL itself may contribute to collagen density. PRL and mammographic density are epidemiologically linked [62, 63] and PRL enhances the expression of mammary ECM components such as *Col1a1* [64], and *Tnc* [65], which promotes cancer cell invasion [66]. Taken together with the current data, these observations begin to outline a model where the characteristics of PRL and ECM work together to promote the invasion and metastasis of hormone-responsive breast cancer.

Mouse models of ERα + breast cancer are limited, and few develop distant metastases [31, 32]. Although patient-derived xenografts are proving useful in elucidating the behavior of some breast cancer subtypes in vivo, they require highly immunocompromised hosts [67, 68], a substantial limitation in light of the accumulating evidence on the importance of the immune response in tumor progression and metastasis [69]. Moreover, ERα + breast tumors have been difficult to grow in mice, preventing the widespread use of patient-derived xenografts for this subtype. Interestingly, mice genetically engineered to express human PRL appear to increase successful transplantations of human primary ERα + breast cancer [70]. Our syngeneic model allows examination of the behavior of ERα + mammary cancers, including metastasis, permitting us to demonstrate potent interactions between the features of the ECM and PRL that drive aggression in vivo.

## Conclusions

Our studies demonstrate that a collagen-I-dense ECM can potently alter hormonal signals to drive the progression of ERα + breast cancer, increasing intravasation and pulmonary metastases. Our data provide evidence that increased collagen-I can shift the pattern of activated signaling cascades in tumor epithelia away from the positive prognostic mediator STAT5 toward pathways that drive poorer outcomes, ERK1/2 and AKT. This is associated with invasive protrusions of the primary tumor that harbor collagen fibers angled perpendicularly to the tumor mass, a hallmark of aggressive ERα + tumors. This remodeling of the surrounding ECM in conjunction with the ability of PRL to increase the expression of mammary ECM components, suggests a feedforward loop by which PRL can drive the metastasis of ERα + cancers.

## Additional files

Additional file 1: Clonal GFP+ TC2GR12 (TC2) and TC4GR5 (TC4) cells are ERα+, PRLR+, and express the signal mediators, STAT5, AKT, and ERK1/2. **A** Wild-type FVB/N mammary epithelial cells (MECs), parental TC2 and TC4 (pTC2, pTC4), and GFP+ TC2 and TC4 RNA were analyzed by real-time PCR for expression of the rat prolactin (*rPrl)* transgene expressed by NRL-PRL animals. MECs did not express the transgene as expected, and transfection with GFP did not alter transgene expression. *1* = MECs; *2* = parental TC2; *3* = TC2GR12 **(**GFP+ TC2); *4* = parental TC4; *5* = TC4GR5 **(**GFP+ TC4). **B** TC2GR12 (TC2) and TC4GR5 (TC4) cells were grown on tissue culture plastic, serum starved overnight, and protein lysates assayed via immunoblotting with the indicated antibodies. (PDF 96 kb)
Additional file 2: Flow cytometry gating strategy to detect circulating tumor cells. **A**-**C** Cultured TC cell lines were trypsinzed, fixed in 2% PFA, and analyzed via flow cytometry. Gates were set to include size of the cells (**A**), inclusion of APC auto-fluorescence (**B**), and selection of GFP+ cells (**C**). **D**-**F** Cleared blood samples were labelled with APC-conjugated CD31, CD45 to mark hematopoietic cells and analyzed following the gating strategy in A-C. Selection for tumor epithelial size (**D**), APC-negative cells (**E**), and GFP+ circulating tumor epithelial cells (**F**). Representative gating strategy shown. (PDF 377 kb)
Additional file 3: Conditions for immunohistochemical and immunofluorescence antibodies. (PDF 256 kb)
Additional file 4: The mCol1a1 environment does not alter tumor PRLR expression, and tumors express progesterone receptors in localized foci. **A** TC tumors lysates were probed for PRLR. No significant differences were observed between WT and mCol1a1 tumors. Mean ± SEM TC2 tumors: WT n = 7, mCol1a1 n = 6; TC4 tumors: WT n = 7, mCol1a1 n = 8. (Mann-Whitney *U* test, *p* > 0.05). **B** Progesterone receptor immunohistochemical analysis. Progesterone receptor is expressed in localized foci (*upper panel*). Positive staining of ductal epithelium (*lower panel*). Original magnifications × 200. *Scale bar* = 50 μm. (PDF 333 kb)
Additional file 5: Surface lung nodules. Lungs from animals with no tumor cell transplants, and those from tumor bearing WT, and mCol1a1 animals. *Arrows* depict surface lung nodules observed under dissection microscopy. Representative images. *Scale bar* = 2 mm. (PDF 434 kb)
Additional file 6: mCol1a1 reduces pSTAT5, and elevates pERK1/2 and pAKT in TC4 tumors, shown by immunohistochemical analysis. Immunohistochemical analysis of pSTAT5 (**A**), pERK1/2 (**B**), and pAKT S473 (**C**) of TC4 tumors. Original magnification × 200. *Scale bar* = 50 μm. (PDF 473 kb)

## Abbreviations

ABC: avidin-biotin complex
APC: allophycocyanin mCol1a1: c*ol1a1* ^*tm1Jae/+*^
CTC: circulating tumor cells
ECM: extracellular matrix
ERK: extracellular regulated kinase
ERα: estrogen receptor alpha
FAK: focal adhesion kinase
FBS: fetal bovine serum
FOV: field of view
GFP: green fluorescent protein
JAK: Janus kinase
NRL: neu-related lipocalin
PBS: phosphate-buffered saline
PRL: prolactin
SFK: src family kinase
SHG: second harmonic generation
STAT: signal transducer and activator of transcription
TC: tumor cell line
WT: wildtype FVB/N mice
