# Supplementary figures and images for: Elevated collagen-I augments tumor progressive signals, intravasation and metastasis of prolactin-induced estrogen receptor alpha positive mammary tumor cells
Source: Breast Cancer Res. 2017 Jan 19;19:9. doi: 10.1186/s13058-017-0801-1 (PMC5244528; doi:10.1186/s13058-017-0801-1)

Barcus et al., Additional File 1

**A**

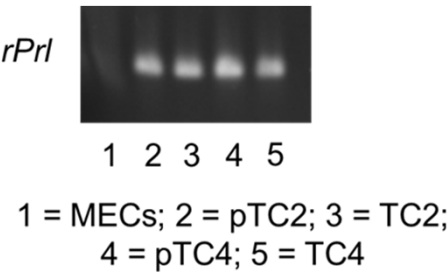

**B**

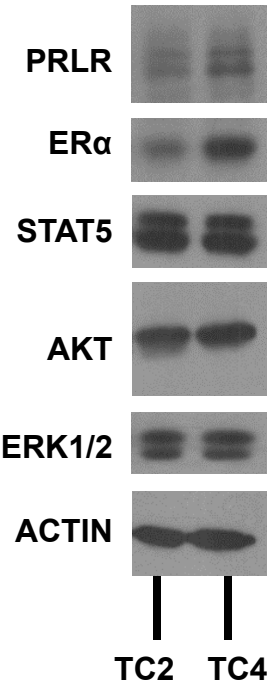

Supplement: Additional file 1: — Clonal GFP+ TC2GR12 (TC2) and TC4GR5 (TC4) cells are ERα+, PRLR+, and express the signal mediators, STAT5, AKT, and ERK1/2. A Wild-type FVB/N mammary epithelial cells (MECs), parental TC2 and TC4 (pTC2, pTC4), and GFP+ TC2 and TC4 RNA were analyzed by real-time PCR for expression of the rat prolactin (rPrl) transgene expressed by NRL-PRL animals. MECs did not express the transgene as expected, and transfection with GFP did not alter transgene expression. 1 = MECs; 2 = parental TC2; 3 = TC2GR12 (GFP+ TC2); 4 = parental TC4; 5 = TC4GR5 (GFP+ TC4). B TC2GR12 (TC2) and TC4GR5 (TC4) cells were grown on tissue culture plastic, serum starved overnight, and protein lysates assayed via immunoblotting with the indicated antibodies. (PDF 96 kb) [file 13058_2017_801_MOESM1_ESM.pdf]

## Barcus et al., Additional File 2

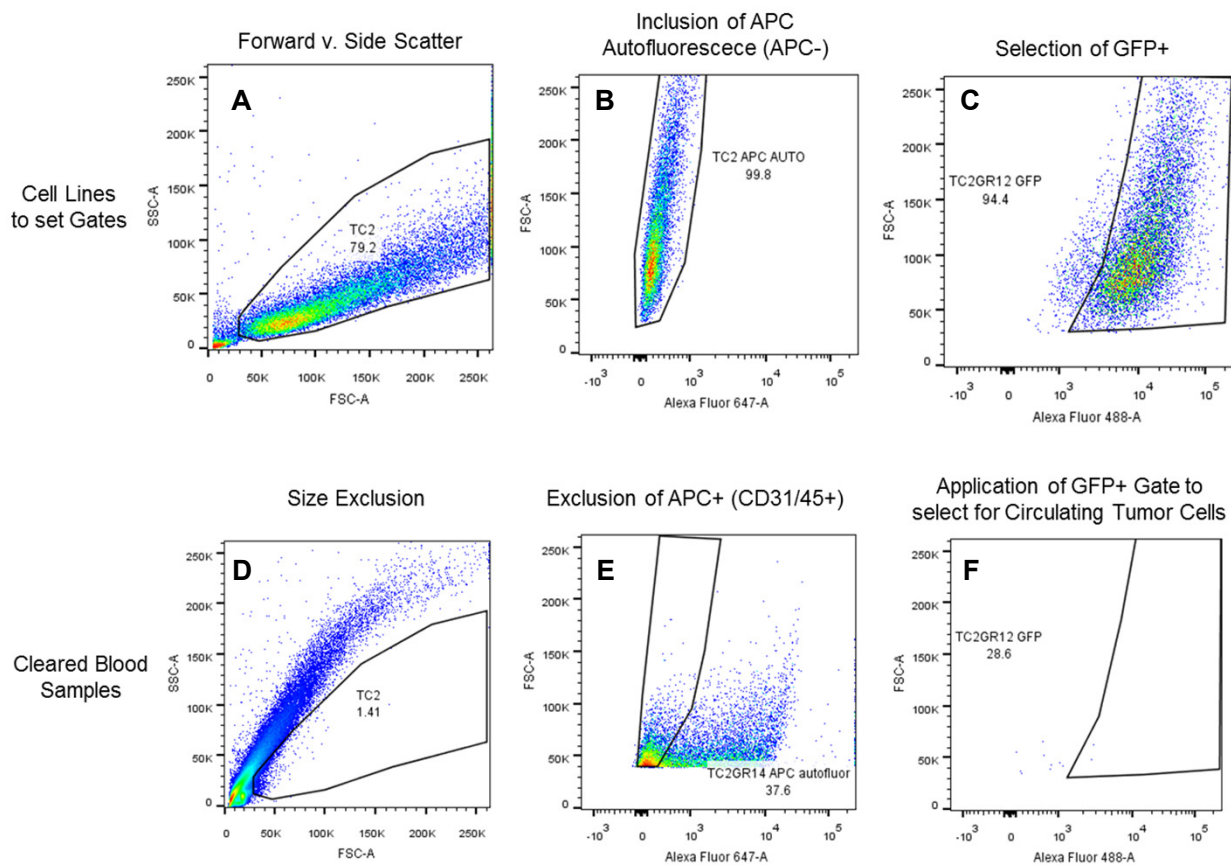

Supplement: Additional file 2: — Flow cytometry gating strategy to detect circulating tumor cells. A-C Cultured TC cell lines were trypsinzed, fixed in 2% PFA, and analyzed via flow cytometry. Gates were set to include size of the cells (A), inclusion of APC auto-fluorescence (B), and selection of GFP+ cells (C). D-F Cleared blood samples were labelled with APC-conjugated CD31, CD45 to mark hematopoietic cells and analyzed following the gating strategy in A-C. Selection for tumor epithelial size (D), APC-negative cells (E), and GFP+ circulating tumor epithelial cells (F). Representative gating strategy shown. (PDF 377 kb) [file 13058_2017_801_MOESM2_ESM.pdf]

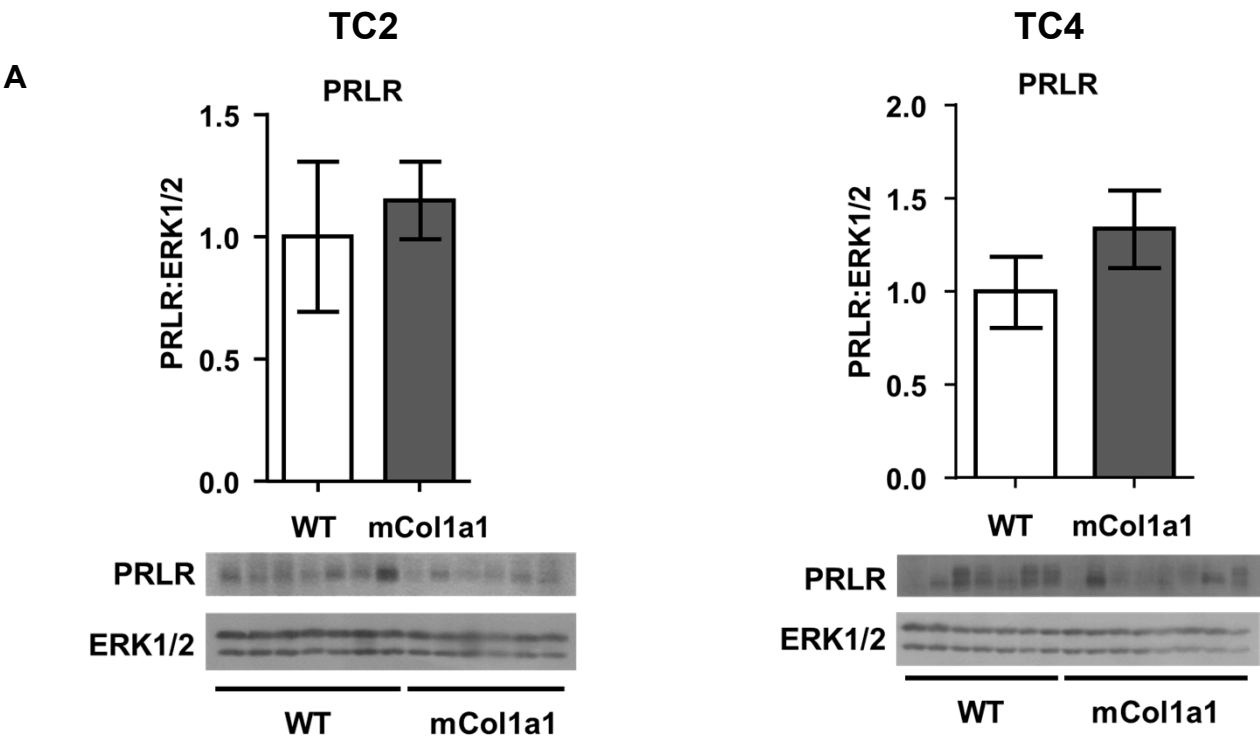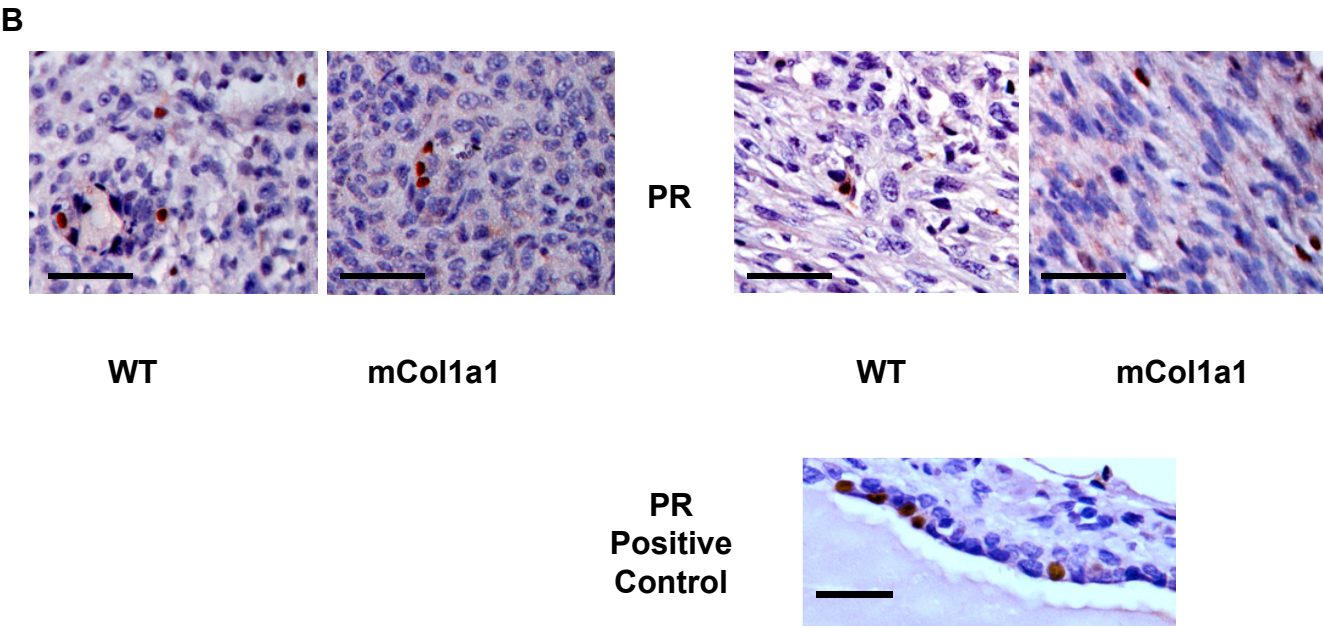

Supplement: Additional file 4: — The mCol1a1 environment does not alter tumor PRLR expression, and tumors express progesterone receptors in localized foci. A TC tumors lysates were probed for PRLR. No significant differences were observed between WT and mCol1a1 tumors. Mean ± SEM TC2 tumors: WT n = 7, mCol1a1 n = 6; TC4 tumors: WT n = 7, mCol1a1 n = 8. (Mann-Whitney U test, p > 0.05). B Progesterone receptor immunohistochemical analysis. Progesterone receptor is expressed in localized foci (upper panel). Positive staining of ductal epithelium (lower panel). Original magnifications × 200. Scale bar = 50 μm. (PDF 333 kb) [file 13058_2017_801_MOESM4_ESM.pdf]

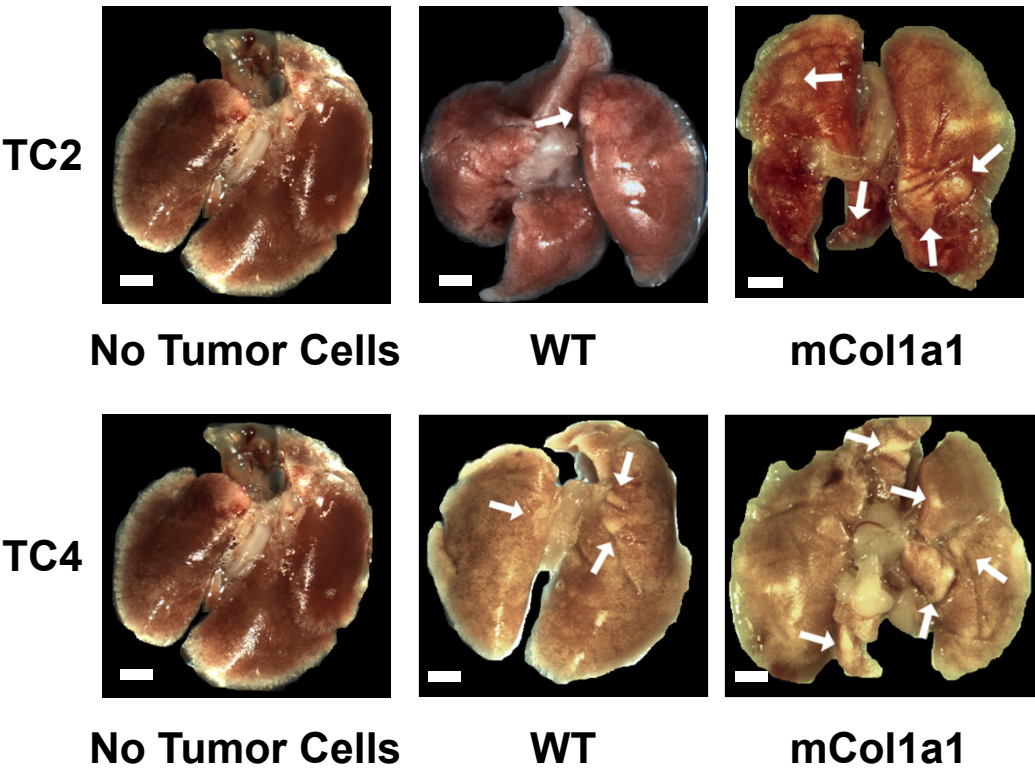

Supplement: Additional file 5: — Surface lung nodules. Lungs from animals with no tumor cell transplants, and those from tumor bearing WT, and mCol1a1 animals. Arrows depict surface lung nodules observed under dissection microscopy. Representative images. Scale bar = 2 mm. (PDF 434 kb) [file 13058_2017_801_MOESM5_ESM.pdf]

Barcus et al., Additional File 6

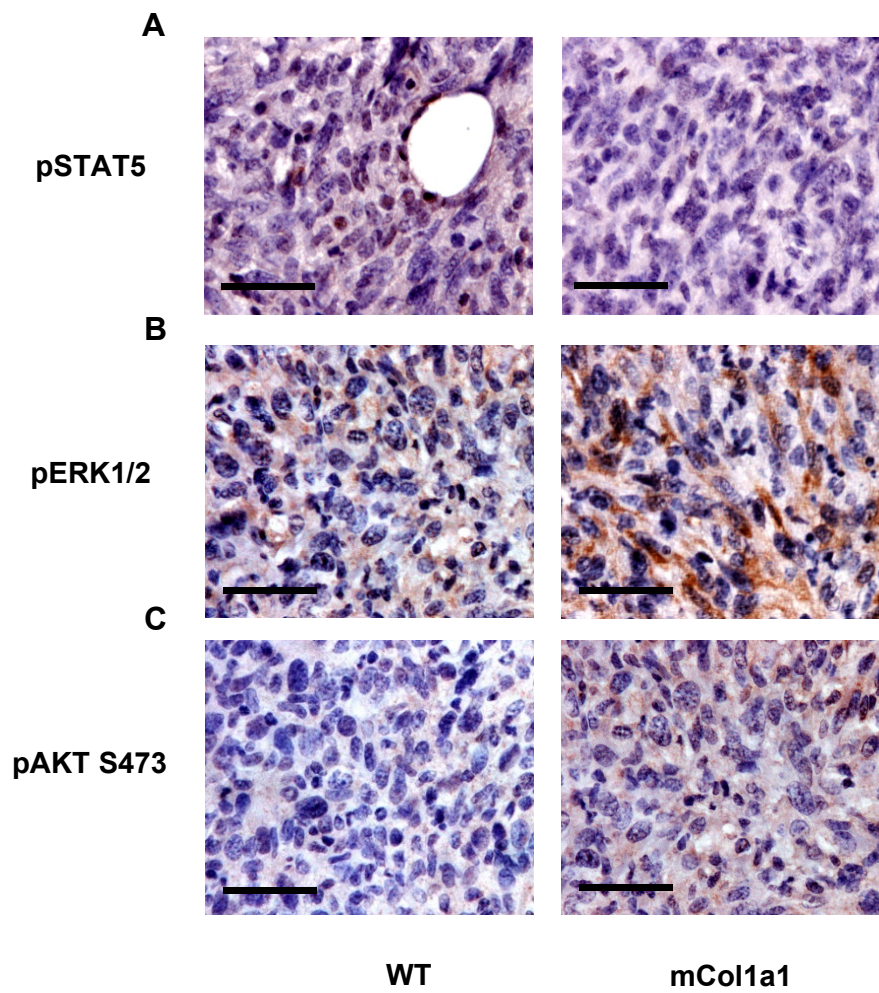

Supplement: Additional file 6: — mCol1a1 reduces pSTAT5, and elevates pERK1/2 and pAKT in TC4 tumors, shown by immunohistochemical analysis. Immunohistochemical analysis of pSTAT5 (A), pERK1/2 (B), and pAKT S473 (C) of TC4 tumors. Original magnification × 200. Scale bar = 50 μm. (PDF 473 kb) [file 13058_2017_801_MOESM6_ESM.pdf]
