# Supplementary material for: Elevated collagen-I augments tumor progressive signals, intravasation and metastasis of prolactin-induced estrogen receptor alpha positive mammary tumor cells
Source: Breast Cancer Res. 2017 Jan 19;19:9. doi: 10.1186/s13058-017-0801-1 (PMC5244528; doi:10.1186/s13058-017-0801-1)
Supplement: Additional file 3: — Conditions for immunohistochemical and immunofluorescence antibodies. (PDF 256 kb) [file 13058_2017_801_MOESM3_ESM.pdf]

Barcus et al., Additional File 3

Additional File 3: Conditions for immunohistochemistry and immunofluorescence antibodies

Immunohistochemical Antigen/Antibody Conditions			
**Antibody**	**Antigen Retrieval**	**Blocking**	**Antibody Concentration**
ERα	Citrate pH 6.0	5% goat serum	1:500
Progesterone Receptor	Citrate pH 6.0	5% goat serum	1:500
pERK1/2	Citrate pH 6.0	5% goat serum	1:400
pSTAT5	Citrate pH 6.0	5% goat serum	1:100
pAKT S473	Citrate pH 6.0	5% goat serum	1:50
Biotinylated-GFP	Citrate pH 6.0	5% goat serum	1:70
